# Largely preserved functionality after the combined loss of NKG2D, NCR1 and CD16 demonstrates the remarkable plasticity of NK cell responsiveness

**DOI:** 10.3389/fimmu.2023.1191884

**Published:** 2023-07-13

**Authors:** Vanna Imširović, Maja Lenartić, Felix M. Wensveen, Bojan Polić, Vedrana Jelenčić

**Affiliations:** Department of Histology and Embryology, Faculty of Medicine, University of Rijeka, Rijeka, Croatia

**Keywords:** NK cells, NKG2D, NCR1, CD16, NK cell activation, innate immunity

## Abstract

Natural killer (NK) cells play an important role in the early defense against tumors and virally infected cells. Their function is thought to be controlled by the balance between activating and inhibitory receptors, which often compete for the same ligands. Several activating receptors expressed on virtually all NK cells lack an inhibitory partner, most notably CD16, NCR1 and NKG2D. We therefore hypothesized that a signal through at least one of these receptors is always required for full NK cell activation. We generated animals lacking all three receptors (TKO) and analyzed their NK cells. *In vitro*, TKO NK cells did not show reduced ability to kill tumor targets but displayed hyperresponsiveness to NK1.1 stimulation. *In vivo*, TKO animals had a minor reduction in their ability to control non-hematopoietic tumors and cytomegalovirus infection, which was the result of reduced NK cell activity. Together, our findings show that activating NK cell receptors without an inhibitory partner do not provide a ‘master’ signal but are integrated in the cumulative balance of activating and inhibitory signals. Their activity is controlled through regulation of the responsiveness and expression of other activating receptors. Our findings may be important for future development of NK cell-based cancer immunotherapy.

## 1 Introduction

Natural killer (NK) cells are cytotoxic cells with an important role in the early defense against intracellular pathogens and tumors (1, 2). NK cells express an array of different germline encoded activating and inhibitory receptors through which they can detect and eliminate potentially dangerous cells. It is believed that the balance of signals received through these receptors controls whether an NK cell gets activated (3, 4). If signals received through inhibitory receptors prevail, the NK cell will stay inactive (5, 6). Inhibitory signals are thought to be surmounted by one of two different ways: 1. A loss of inhibitory signals, such as a reduction of MHC-I expression. This process typically occurs after viral infection or oncogenic transformation and is referred to as ‘missing self’ (7). 2. Through increased engagement of activating receptors. These molecules recognize ligands whose expression is induced or enhanced in target cells after infection or oncogenic transformation (“induced self” and “non-self”). Sufficient expression of activating ligands is thought to shift the balance of signals towards activation (8, 9). However, whether additional modes of NK cell activation are possible is still unclear.

NK cells are potent cellular weapons in the fight against tumors, but one of the biggest obstacles to harnessing them therapeutically is our incomplete understanding of NK cell activation. This is especially important since inappropriate activation of cytotoxic cells can cause tissue damage or can even lead to autoimmune disease. For this reason, T cells require co-stimulation and cytokines in addition to the ‘master’ activating signal through their T cell receptor (TCR) to prevent inappropriate activation (10). To ensure proper immune-surveillance, whilst preventing auto-immune disease, also NK cell activation needs to be tightly regulated. This raises the question whether the balance model, in which activating signals needs to prevail over inhibitory signals, is sensitive enough. One key group of activating NK cell receptors made up by NKG2D, CD16 and NKp46 (NCR1 in mice), significantly deviates from all others, both in the way they activate NK cells and the way through which their activation threshold is determined. CD16 plays a key role in antibody-dependent cell cytotoxicity (ADCC) (11), whereas NKG2D and NKp46 are activated through induced self-ligands (3, 12, 13). In contrast to most other NK cell receptors, these three molecules are expressed on all NK cells and lack an inhibitory partner competing for the same ligands. Therefore, the ability of these ‘non-paired’ receptors to activate NK cells may be less dependent on shifts in the overall activation state of NK cells. Instead, the activation threshold of these receptors appears to be mediated by the activity of other non-paired receptors (14). However, whether the engagement of these molecules is required for NK cell activation as a ‘master signal’ similar to the TCR in T cells is currently unknown.

We created mice simultaneously lacking the NKG2D, NCR1 and CD16 receptors (TKO). TKO mice showed an impaired ability to control non-hematopoietic tumors and cytomegalovirus infection. However, their ability to produce cytokines and mediate tumor target cell killing *in vitro* was not greatly reduced. Analysis of single knock out mice revealed that lack of just one of these activating receptors causes changes of NK cell receptor repertoire as well as receptor-specific hyperreactivity. Thus, whereas non-paired activating receptors play an important role in NK cell mediated control of cellular threats, their engagement is not absolutely required for NK cell activation. Our findings provide deeper insights into the plasticity of NK cell responsiveness, which may be important for the future development of NK cell-based cancer immunotherapy.

## 2 Results

### 2.1 Triple deficiency of CD16, NKG2D and NCR1 has a minor impact on NK cell development

**Figure 1:**
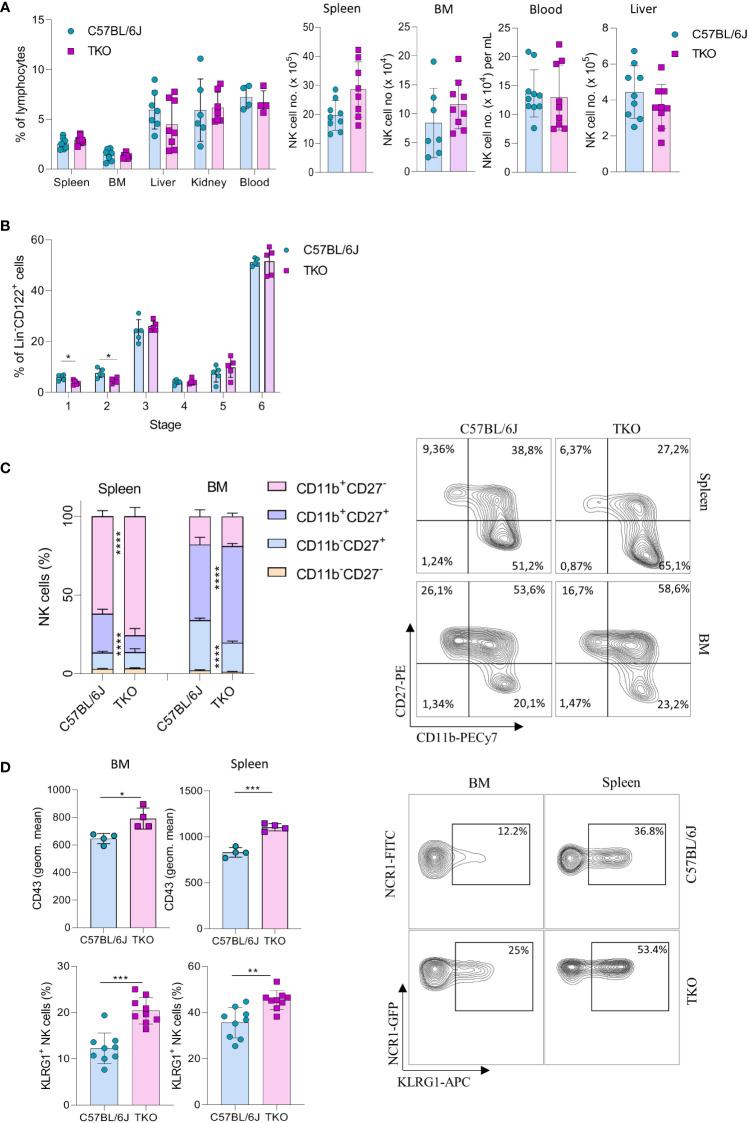
Lack of CD16, NKG2D and NCR1 has mild influence on NK cell development. **(A)** Percentages (left) and total numbers (right) of NK cells (gated CD3^-^NK1.1^+^NCR1^+^) in spleen (n=8-9 mice per group), bone marrow (BM) (n=8-9 mice per group), blood (n=9-10 mice per group), liver (n=9 mice per group) and kidney (n= 6-7 mice per group) (gated CD3^-^NK1.1^+^NCR1^+^Eomes^+^CD49a^-^) from WT (C57BL/6J) and *FcγR3a* ^-/-^ *Klrk1* ^-/-^ *Ncr1* ^gfp/gfp^ (TKO) mice. **(B)** NK developmental stages in the BM; Stage 1 (CD122^+^NK1.1^-^NCR1^+^CD11b^-^c-kit^-^), stage 2 (CD122^+^NK1.1^+^NCR1^-^CD11b^-^c-kit^+^), stage 3 (CD122^+^NK1.1^+^NCR1^+^CD11b^-^c-kit^-^), stage 4 (CD122^+^NK1.1^+^NCR1^+^CD11b^-^c-kit^+^), stage 5 (CD122^+^NK1.1^+^NCR1^+^CD11b^+^c-kit^-/+^), stage 6 (CD122^+^NK1.1^+^NCR1^+^CD11b^+^c-kit^-^) (n = 5 mice per group). **(C)** Representative flow cytometry plots and percentages of CD11b, CD27 expression on NK cells in the spleen and BM of WT and TKO mice (n = 10 mice per group). **(D)** Representative flow cytometry plots, geometric mean of CD43^+^ and percentages of KLRG1^+^ NK cells in the spleen and BM (n = 9 mice per group). Each symbol represents an individual mouse. Data shown represent pooled data from two independent experiments **(A, C, D)** or one representative experiment out of 3 independent experiments **(B)**. Mean ± SD is shown for the presented data. *p < 0.05, **p < 0.01, ***p < 0.001 and ****p < 0.0001. Unpaired Student’s t-tests (two-tailed) **(A, B, D)** or Two-way ANOVA **(C)** were used to calculate these values.

To investigate the role of CD16, NKG2D and NCR1 in NK cell activation we generated ‘triple knockout’ (TKO) mice, i.e. animals with genetic deficiency for all three receptors. TKO mice were healthy and did not show changes in the frequency of B cells, CD4^+^ and CD8^+^ T cells compared to WT controls (**Supplementary Figure 1A**). In addition, the frequency and numbers of NK cells in the spleen, bone marrow (BM), liver, kidney and blood were comparable to those in WT controls (**Figure 1A**).

Next, we wanted to investigate whether the lack of these three activating receptors influences NK cell development. In the bone marrow we only observed minor changes in maturation, with a decrease in stage 1 (CD122^+^NK1.1^-^NCR1^+^CD11b^-^c-kit^-^) and 2 (CD122^+^NK1.1^+^NCR1^-^CD11b^-^c-Kit) cells (**Figure 1B**). In contrast, when looking at CD27 and CD11b (15), TKO mice had NK cells with a more mature phenotype, both in the spleen and in the bone marrow (**Figure 1C**). To determine whether the change in NK cell maturation depended on an individual receptor, we analyzed the phenotype of NK cells in the spleens of animals lacking only a single gene. The maturation status of NK cells in *Klrk1* ^-/-^ and *Ncr1* ^gfp/gfp^ mice was not altered compared to WT mice, whereas animals deficient for FcγRIIIa *(Fcgr3a* ^-/-^) displayed a reduced frequency of late-stage NK cells (**Supplementary Figure 1B**). In the bone marrow, lower percentages of CD11b^-^CD27^+^ and a concomitant increase of CD11b^+^CD27^+^ cells were observed (**Figure 1C**). This implies that early maturation is the same but that signals in the periphery (and lesser extent BM) drive late maturation.

To investigate whether other markers of late maturation are affected by triple deficiency of non-paired receptors, we investigated expression of CD43 and KLRG1 (16, 17). We observed a significant increase in both markers on NK cells in the spleen and BM of TKO mice (**Figure 1D**). The difference in KLRG1 expression was not observed in any of the single knockout mice (**Supplementary Figure 1C**).

Taken together, these data indicate that the lack of CD16, NKG2D and NCR1 has only a minor, but synergistic impact on NK cell development. Their deficiency results in a more mature phenotype of NK cells in the periphery, whereas it does not affect NK cell numbers and frequency.

### 2.2 CD16/NKG2D/NCR1 triple deficiency causes changes in the NK cell receptor repertoire

**Figure 2:**
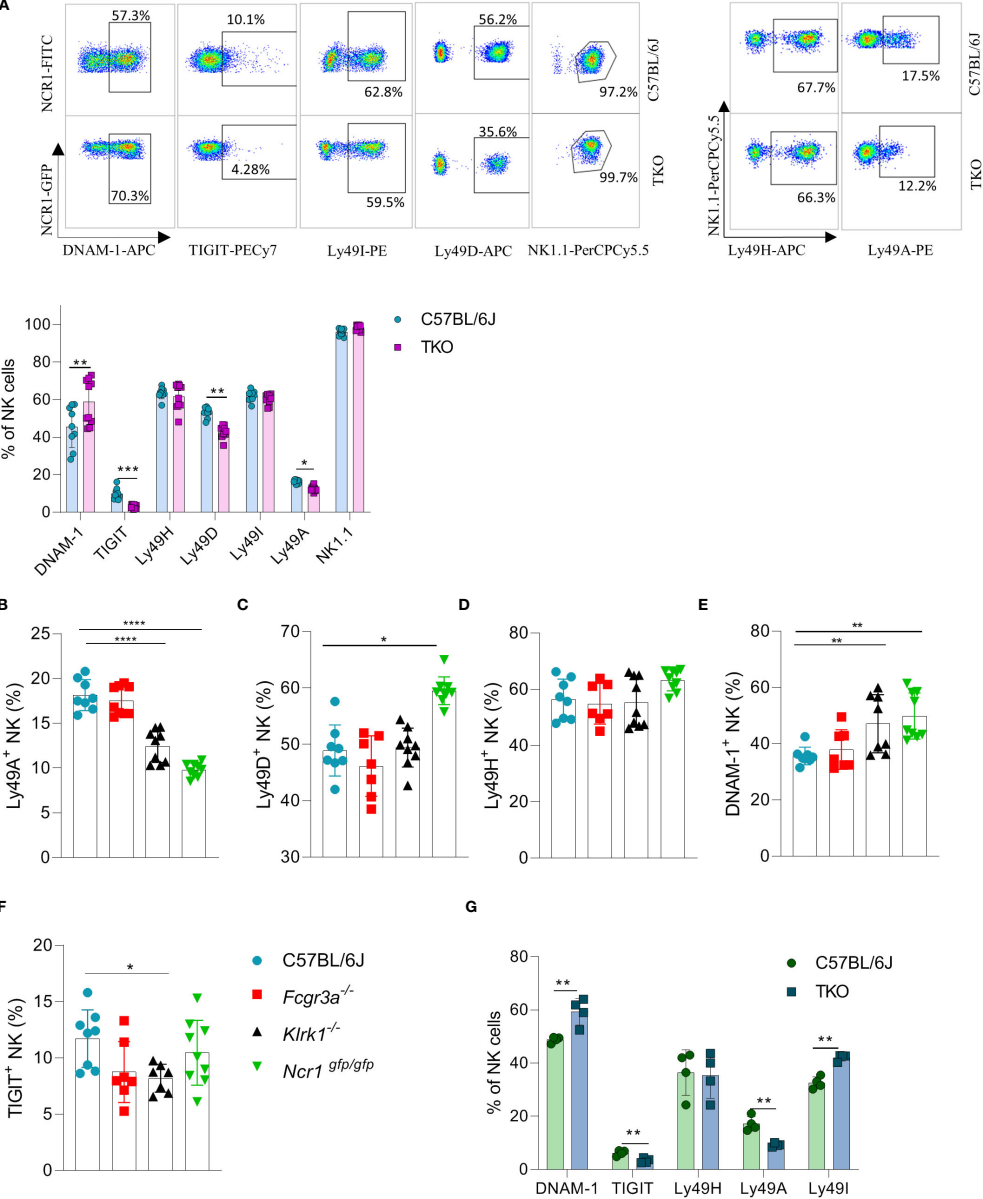
Differences in NK cell receptor repertoire in mice lacking NKG2D, NCR1 and CD16 receptors. **(A)** Representative FACS plots of the expression of DNAM-1, TIGIT, Ly49I, Ly49A, Ly49H and NK1.1 from spleens of WT and TKO mice. Percentages of NK cells expressing DNAM-1, TIGIT, Ly49H, Ly49D, Ly49I, Ly49A from WT (n = 6-9), TKO (n = 10), *FcγR3a* ^-/-^ (n = 7), *Klrk1* ^-/-^ (n = 9) and *Ncr1* ^gfp/gfp^ mice (n = 9) **(A–F)** or mixed bone marrow chimeras (n = 4) **(G)**. Each symbol represents an individual mouse. Data shown represent pooled data from two independent experiments(A-F) or one representative experiment out of 2 independent experiments **(G)**. Mean ± SD is shown for the presented data. *p < 0.05, **p < 0.01, ***p < 0.001 and ****p < 0.0001. Unpaired Student’s t-tests (two-tailed) **(A, G)** and One-way ANOVA **(B–F)** were used to calculate these values.

We previously observed that the lack of one NK cell receptor may impact the expression of others. We therefore analyzed the expression profile of other activating and inhibitory NK cell receptors. We found that several Ly49 receptors were downregulated, including Ly49D and Ly49A (**Figure 2A**). To investigate whether the differential control of Ly49 receptors depended on the synergistic loss of three non-paired receptors, we investigated their expression on animals lacking only a single gene. A similar downregulation of Ly49A was observed in *Klrk1* ^-/-^ and *Ncr1* ^gfp/gfp^ mice, but not in *Fcgr3a* ^-/-^ mice (**Figure 2B**). No differences were seen in Ly49D expression on NK cells of *Klrk1* ^-/-^ and *Fcgr3a* ^-/-^ mice compared to WT mice, whereas there was significant upregulation of this receptor in *Ncr1* ^gfp/gfp^ mice (**Figure 2C**). The expression levels of Ly49H were similar across the genotypes (**Figure 2D**).

Interestingly, a higher expression of DNAM-1 was observed in TKO mice (**Figure 2A**). In contrast, TIGIT, an inhibitory receptor that competes with DNAM-1 for binding to PVR (18), was found downregulated in TKO mice (**Figure 2A**). Upregulation of DNAM-1 was also seen in *Klrk1* ^-/-^ and *Ncr1* ^gfp/gfp^ mice (**Figure 2E**) but was not associated with a concomitant decrease in TIGIT (**Figure 2F**). Deficiency of CD16 did not impact expression of DNAM-1 or TIGIT. Thus, whereas loss of individual activating receptors impacts the DNAM-1/TIGIT balance, deficiency of all three has the strongest effect.

CD16 is known to impact functional properties of macrophages and dendritic cells (DC) (11, 19). To determine whether the observed differences in receptor expression were cell intrinsic, we generated mixed bone marrow chimeras in which TKO and WT bone marrow cells were mixed in a 1:1 ratio and transferred to irradiated mice with different congenic markers. Differences in receptor expression observed in mixed bone marrow chimeras corresponded with the ones seen in the TKO mice (**Figure 2G**).

Our findings show that the surface expression of individual receptors is fine-tuned by the presence of others. This suggests that a compensating mechanism exists to maintain the activation equilibrium in NK cells even in the absence of three major activating receptors.

### 2.3 TKO NK cells show specific hyper-responsiveness after stimulation through NK1.1 *in vitro*

**Figure 3:**
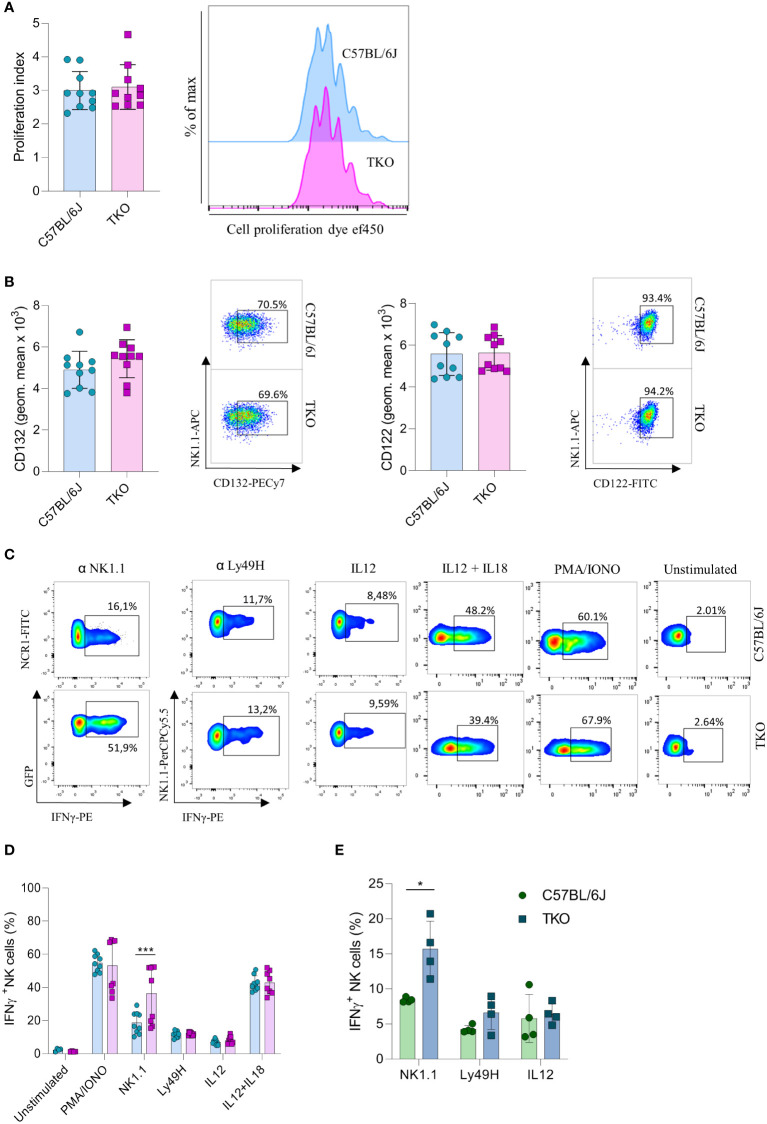
Differences in NK cell responsiveness after *in vitro* stimulation in mice lacking NKG2D, NCR1 and CD16 receptors. **(A)** Enriched NK cells from WT and TKO mice were stimulated with IL15 and after 4 days in culture proliferation index was analyzed (left) with representative histograms (right) (n = 10 mice per group). **(B)** Geometric mean and representative FACS plots of CD122^+^ and CD132^+^ NK cells after IL15 stimulation 4 days in culture (n = 10 mice per group). **(C)** Representative FACS plots gated for CD3^−^NCR1^+^ cells (NK1.1 stimulation) or CD3^−^NK1.1^+^ cells (other stimuli) in WT and TKO mice. NK cells from WT and TKO mice (n = 8-10 mice per group) **(D)** or mixed bone marrow chimeras (n = 4 mice per group) **(E)** were stimulated for 4 h through NK1.1 and Ly49H receptor by mAb or with PMA/IONO, IL12, IL12+IL18 and IFN-γ production was analyzed. Each symbol represents an individual mouse. Data shown represent pooled data from two independent experiments **(A–D)** or one representative experiment out of 2 independent experiments **(E)**. Mean ± SD is shown for the presented data. *p < 0.05 and ***p < 0.001. Unpaired Student’s t-tests (two-tailed) was used to calculate these values.

Our findings indicate that the lack of activating receptors is compensated by modifying the expression of others. To investigate whether this compensatory effect also operates at a functional level, we performed a series of *in vitro* stimulations to test the responsiveness of TKO NK cells to activating signals. IL-15 is an essential cytokine for the survival and proliferation of NK cells (20). Therefore, splenic NK cells of WT and TKO mice were cultured in the presence of IL-15 and after 4 days their proliferation was measured. There was no significant difference in the proliferation capacity of TKO and WT NK cells (**Figure 3A**). The expression levels of CD132 and CD122, subunits of the IL-15 receptor, were also comparable to those in WT controls (**Figure 3B**). These data indicate that lack of non-paired activating receptors does not influence the proliferation capacity of NK cells in response to IL-15.

We next investigated the responsiveness of other activating receptors in NK cells of TKO mice. When stimulated through NK1.1, TKO NK cells showed a significantly higher production of IFNγ compared to the WT NK cells (**Figures 3C, D**). This effect was also seen in CD16 and NKG2D deficient mice, but not in animals lacking NCR1 (**Supplementary Figure 2A**). Differences in responsiveness were not observed following Ly49H, IL-12 or IL-12 plus IL-18 stimulation, as production of IFNγ was comparable to those of WT NK cells (**Figures 3C, D**). To determine whether the observed effects were indeed NK cell intrinsic, we performed NK1.1, Ly49H and IL-12 stimulation of NK cells isolated from mixed bone marrow chimeras (**Figure 3E**). The NK1.1 hyper-responsiveness was also observed in the chimeric system, whereas the sensitivity of other receptors was not affected by receptor triple-deficiency. Stimulation of TKO NK cells with the receptor-bypassing stimulus, phorbol-12-myristate-13-acetate (PMA) plus ionomycin did not result in differences in IFNγ production, indicating that modulation of NK cell responsiveness operates at the receptor level and not in the downstream signaling machinery, while differences were observed in CD16 and NKG2D deficient mice (**Figures 3C, D**, **Supplementary Figure 2B**).

Thus, NK cells of mice lacking three non-paired activating receptors have altered sensitivity to other activating NK cell receptors in a cell-intrinsic fashion.

### 2.4 CD16, NKG2D and NCR1 are dispensable for the cytolytic activity of NK cells *in vitro*

**Figure 4:**
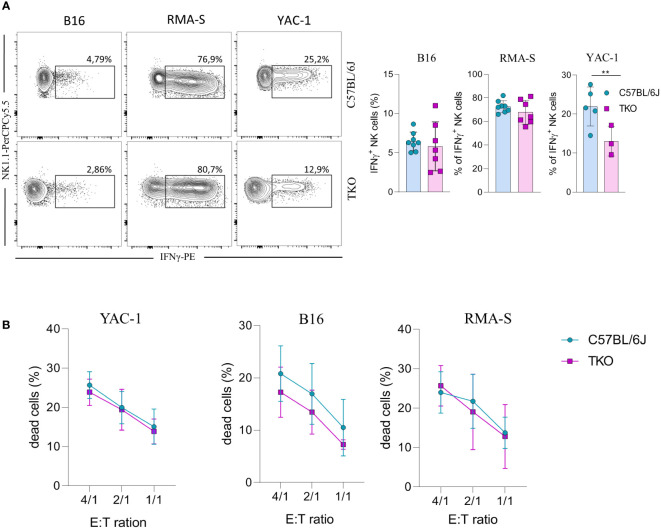
Responsiveness of NK cells deficient for NKG2D, NCR1 and CD16 receptors to hematopoietic and non-hematopoietic tumor targets *in vitro*. **(A)** Spleenocytes from indicated mice were stimulated with tumor target cells in 1:1 ratio. Percentages of IFN-γ^+^ NK cells were analyzed (right panel) and representative flow cytometry plots are enclosed (left) (n = 7-8 mice per group). **(B)** Killing capacity of NK cells from WT and TKO mice (n = 3 mice per group) towards YAC-1/B16/RMA-S-eFlour450 labeled cells after 4h at indicated effector to target ratios. Data shown represent pooled data or one representative experiment out of three independent experiments. Mean ± SD is shown for the presented data. **p < 0.01. Unpaired Student’s t-tests (two-tailed) **(A)** or Two-way ANOVA **(B)** were used to calculate these values.

Since we observed alterations in receptor expression, we wanted to investigate the ability of TKO NK cells to respond to putative hematopoietic (RMA-S) and non-hematopoietic (B16, YAC-1) tumor targets *in vitro*. Splenic NK cells were co-cultured with B16, RMA-S and YAC-1 cells, and their IFNγ production was measured. B16 is a mouse melanoma that expresses NCR1 and DNAM-1 ligands (21–24). RMA-S is a mouse lymphoma cell line that is MHC-I deficient and therefore susceptible to “missing self”-mediated killing (25), while YAC-1 is a mouse lymphoma cell line that expresses NKG2D ligands (26). Only in the case of YAC-1 co-cultivation did we observe a significant decrease in IFNγ production, whereas co-cultivation of TKO cells with RMA-S or B16 cells did not result in a changed production of IFNγ (**Figure 4A**).

We next assessed the cytolytic capacity of TKO NK cells following *in vitro* co-cultivation of these cells with tumor targets. Splenic TKO NK cells killed YAC-1, B16 and RMA-S target cells with an efficiency equal to that of WT NK cells (**Figure 4B**). Thus, the loss of CD16, NKG2D and NCR1 does not cause any defect in the ability of NK cells to kill hematopoietic and non-hematopoietic tumor targets *in vitro.*

### 2.5 TKO mice have a reduced capacity to control non-hematopoietic tumors *in vivo*

**Figure 5:**
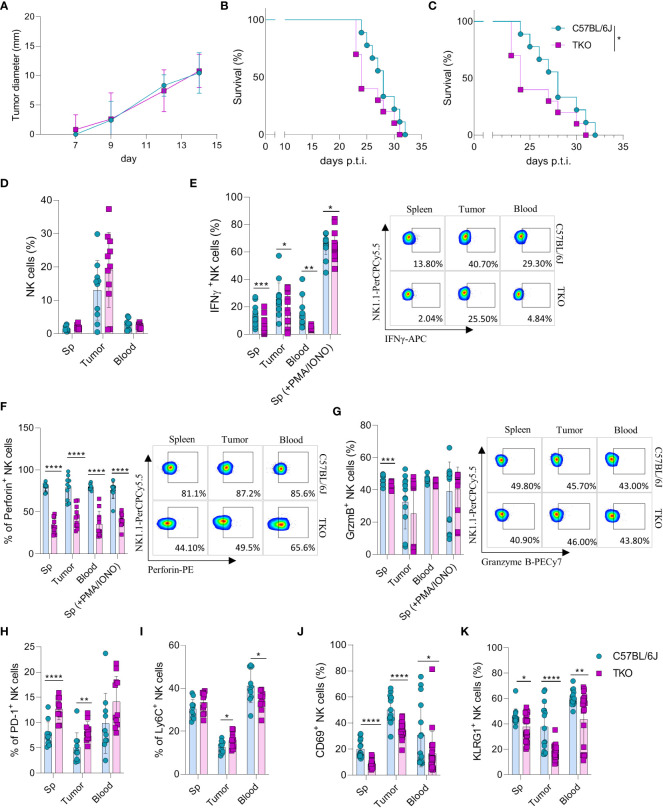
Control of hematopoietic and nonhematopoietic tumors in TKO mice. Graphs shows tumor size **(A)** or survival curve **(B)** of WT and TKO mice (n = 10 mice per group) after injection of RMA-S cells i.v. **(A)** or s.c. **(B, C)** Survival curve of TKO mice and indicated controls (n = 10 mice per group) after i.v. injection of B16 cells. **(D)** Percetages of NK cells in spleen, tumor and blood of WT and TKO mice (n = 10-11 mice per group). WT or TKO (n = 15-21 mice per group) NK cells from the spleen and s.c. B16 tumor were left in culture for 4h or stimulated with PMA/IONO and IFNγ **(E)**, Perforin **(F)** or Granzyme B production **(G)** was analyzed. **(H–K)** Percentages of NK cells expressing PD-1, Ly6C, CD69 and KLRG1 isolated from spleen, blood and B16 s.c. tumor of WT and TKO mice. Each symbol represents an individual mouse. Data shown represent one out of two independent experiments **(A–C)** or pooled two independent experiments **(D-K)**. Mean ± SD is shown for the presented data. *p < 0.05, **p < 0.01, ***p < 0.001 and ****p < 0.0001. Survival curves were analyzed by the Kaplan-Meier model followed by log-rank (Mantel-Cox) test (two-tailed). Unpaired Student’s t-tests (two-tailed) **(D–K)** was used to calculate these values.

Our findings indicate that NKG2D, NCR1 and CD16 are not required for the activation of NK cells *in vitro*. However, we hypothesized that their role may be indispensable for their functionality *in vivo*. Therefore, RMA-S cells were injected in WT or TKO mice and tumor progression or survival of animals was followed. We did not observe any differences in tumor growth after s.c. injection of RMA-S cells (**Figure 5A**), nor was survival of animals affected in TKO mice (**Figure 5B**). Next, we examined the ability of these cells to control B16 melanoma cells. Mice were injected i.v. with B16 cells and survival was followed. Compared with WT controls, TKO mice showed significantly reduced survival rates (**Figure 5C**). Observed differences were due to NCR1 deficiency, as mice lacking only this receptor showed comparable survival to TKO animals (**Supplementary Figure 3**). This goes in line with previously published data on Ncr1-deficient animals (14, 23).

To elucidate why TKO NK cells mediate reduced control of B16 tumor growth, tumor -associated NK cells (TANKs) from tumors of equal size were analyzed and compared with those in the spleen and blood of WT and TKO mice. Although there was no difference in the percentage of infiltrating NK cells (**Figure 5D**), phenotypic alternations were observed. TANKs from TKO mice showed reduced IFNγ production (**Figure 5E**), a cytokine which is known to play a pivotal role in *in vivo* control of B16 melanoma (14). Moreover, reduced IFNγ production was observed in TKO NK cells obtained from the spleen and blood (**Figure 5E**). TKO NK cells also showed significantly reduced levels of Perforin (**Figure 5F**), while Granzyme B production was only slightly reduced (**Figure 5G**). The observed reduction in IFNγ and Perforin production corresponds with the increase in expression of exhaustion markers such as PD-1 and Ly6C in TKO NK cells (**Figures 5H, I**). Notably, the expression level of CD69 and KLRG1 were significantly reduced in the spleen, blood and tumor of TKO animals, indicating that these cells are less activated compared to those in WT controls (**Figures 5J, K**).

Altogether, these data indicate that TKO NK cells are suppressed in the B16 tumor micro-environment compared to WT NK cells. This is most likely the result of NCR1 deficiency, resulting in reduced survival in the B16 tumor model.

### 2.6 *TKO* mice have impaired control of MCMV infection

**Figure 6:**
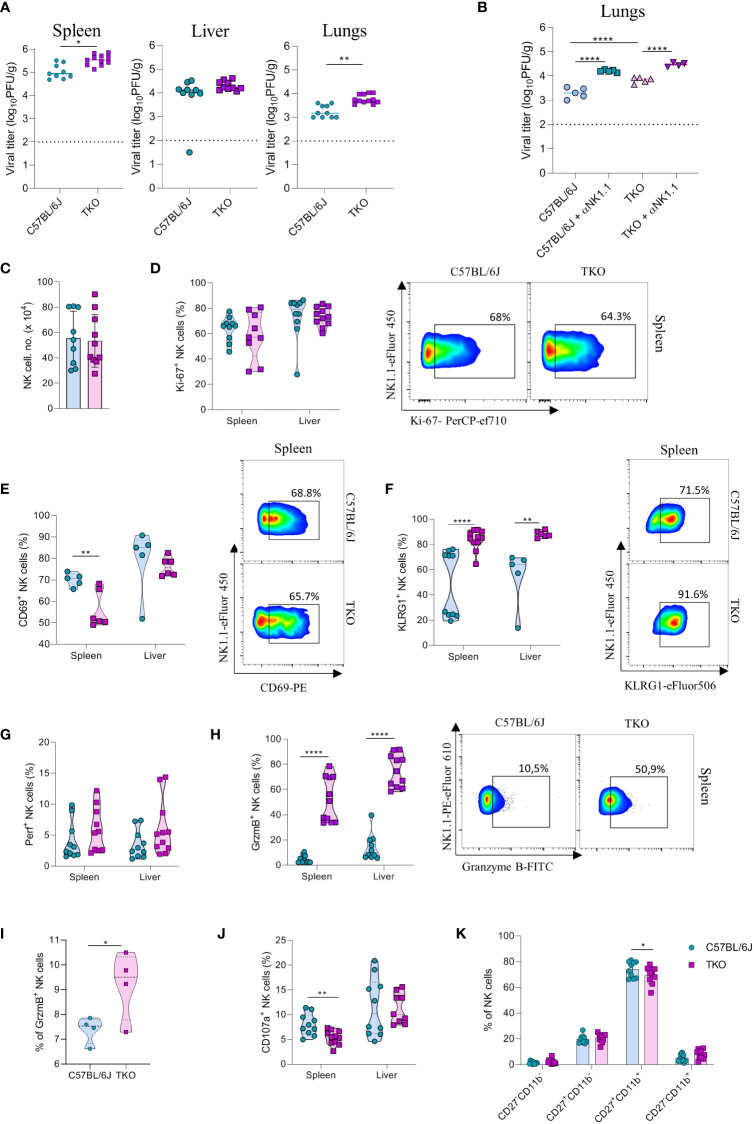
mpaired control of MCMV infection in the absence of NKG2D, NCR1 and CD16 receptors. **(A)** WT and TKO mice (n = 8-12 mice per group) were infected with WT MCMV (2 x 10^5^ PFU intravenously). Viral titers were assessed in the spleen, liver and lungs. **(B)** TKO and WT mice (n = 5 mice per group) were treated with anti-NK1.1 one day before the infection with WT MCMV (2 x 10^5^ PFU intravenously); viral titers were assessed in lungs. **(C)** Total numbers of NK cells in spleen of WT and TKO mice. **(D–F)** Representative FACS plots and percentages of NK cells expressing Ki-67, CD69 and KLRG1 isolated from the spleen and liver of WT and TKO mice. NK cells from the spleen and the liver were left in culture for 4h and Perforin **(G)**, Granzyme B **(H)**, representative plots right) or CD107a **(J)** expression levels were analyzed. **(I)** NK cells from WT and TKO mice (n = 4 mice per group) were stimulated with IFNβ for 4h and Granzyme B production was analyzed. **(K)** Percentages of CD11b and CD27 expression on NK cells in the spleen of WT and TKO mice. Data shown represent pooled data from two independent experiments **(A, C, D, F–J)** or one out of three independent experiments **(B, E)**. Mean ± SD is shown for the presented data. *p < 0.05, **p < 0.01 and ****p < 0.0001. Unpaired Student’s t-tests (two-tailed) **(A, C–I)** or One-way ANOVA **(B, J)** were used to calculate these values.

NK cells are very important for the early control of viral infection (27). To investigate whether receptor triple-deficiency compromises anti-viral responsiveness of NK cells, we infected TKO mice with murine cytomegalovirus (MCMV). At 3.5 days post infection viral titers (PFU) were determined in several organs. Viral titers in the spleen and lungs of TKO mice were significantly higher than those in the control group (**Figure 6A**), whereas the viral titers in the liver were not affected (**Figure 6A**). The increase in lung viral titers was not observed in mice lacking only a single activating receptor (**Supplementary Figure 4A**). NK cell depletion of mice before the infection negated differences between WT and TKO mice, indicating that differences in viral titers are the result of impaired functionality of these cells (**Figure 6B**, **Supplementary Figure 4B**). Nevertheless, NK depletion increased titers both in WT and TKO mice, indicating that deficiency of the three receptors does not abrogate NK cell activity completely.

We next investigated why NK cells of TKO mice have a reduced ability to control MCMV infection. We did not observe changes in the number of NK cells in the spleen after infection (**Figure 6C**). In accordance with this notion, proliferation of cells quantified by Ki-67 staining was comparable between WT and TKO animals (**Figure 6D**). We therefore analyzed the functional capacity of these cells. As in the B16 model, we observed that NK cells of TKO mice have reduced induction of CD69 compared to WT animals (**Figure 6E**). The percentage of KLRG1^+^ NK cells was increased in both the spleen and the liver of TKO mice (**Figure 6F**), as well as TIM-3, TIGIT and LAG-3 (**Supplementary Figure 4D**), suggesting a more exhausted phenotype of these cells. Increased KLRG1 levels were also observed in splenic NK cells from *Klrk1* ^-/-^ and *Ncr1* ^gfp/gfp^ mice, whereas lower induction of CD69 was only seen in the latter genotype (**Supplementary Figure 4C**). Interestingly, whereas Perforin expression was not affected, NK cells of TKO mice expressed significantly higher levels of Granzyme B (**Figures 6G, H**). This upregulation was present both in the spleen and the liver. The same effect was also observed in the liver and spleen of *Ncr1* ^gfp/gfp^ mice, indicating that this phenotype comes from the loss of the NCR1 receptor (**Supplementary Figure 4C**). During viral infection cytokines play an important role in the initial NK cell activation, particularly type I interferons. Indeed, stimulation with IFN-β resulted in an increase of granzyme B production of WT NK cells, which was further increased in TKO cells (**Figure 6I**). Notably, expression of the degranulation marker CD107a was lower on the surface of TKO NK cells, suggesting that Granzyme B accumulates in these cells as a result of a reduced secretion rate (**Figure 6J**). The maturation status of NK cells in TKO mice was predominantly unaltered, with the exception of CD27^+^CD11b^+^ population which was slightly decreased in TKO mice (**Figure 6K**).

**Figure 7:**
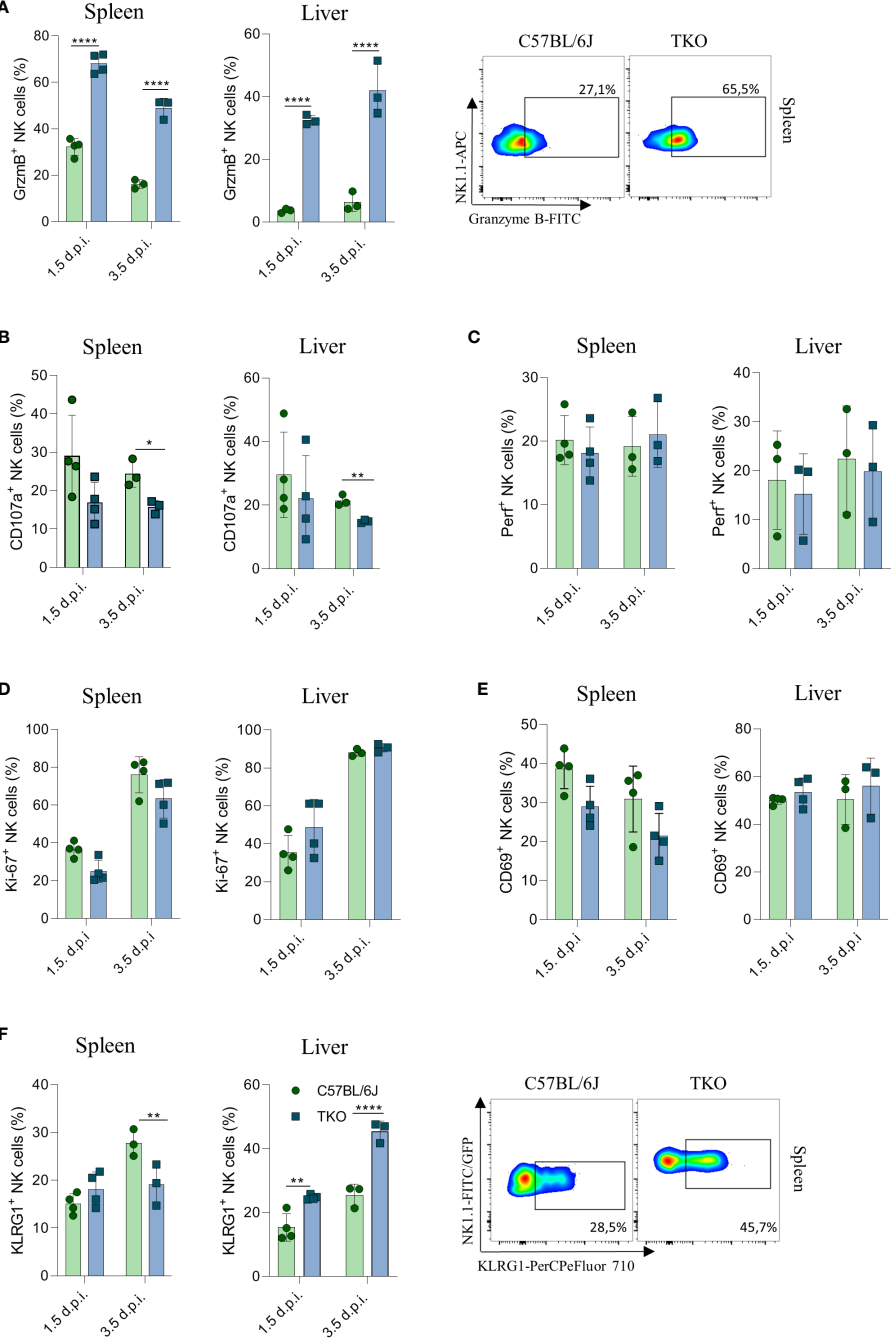
Control of MCMV infections in mixed bone marrow chimeras. Mixed bone marrow chimeras (n = 4) were infected with WT MCMV (2 x 10^5^ PFU intravenously) and analyzed 1.5 and 3.5 days post infection. NK cell were left 4 h in culture and expression levels of Granzyme B **(A)**, representative plots right), CD107a **(B)** and Perforin **(C)** were analysed in the spleen and liver. **(D–F)** Percentages of NK cells expressing Ki-67, CD69 and KLRG1 (representative plots right) in the spleen and liver of WT an TKO mice. Data shown represent one out of two independent experiments. Mean ± SD is shown for the presented data. *p < 0.05,**p < 0.01 and ****p < 0.0001. Unpaired Student’s t-tests (two-tailed) was used to calculate these values.

To confirm that the altered response of TKO NK cells following MCMV infection are indeed cell intrinsic, mixed bone marrow (BM) chimeras were generated in which WT and TKO tissue was transplanted in wild type recipients. Chimeras were infected with MCMV and analyzed at 1.5- and 3.5-days post infection. The upregulation of Granzyme B and downregulation of CD107a expression were also present in the chimeric system (**Figures 7A, B**), whereas Perforin, Ki-67 and CD69 expression levels remained unchanged between groups (**Figures 7C–E**). Increased KLRG1 expression was observed in NK cells from TKO mice isolated from the liver, but not from the spleen (**Figure 7F**).

Our results demonstrate that lack of all the three activating receptors results in a compromised control of MCMV infection compared to WT mice causing higher viral titers in spleen and lungs, possibly as a result of impaired degranulation.

## 3 Discussion

In the present study we investigated the provocative hypothesis that the current model for NK cell activation based on a balance between inhibitory and activating signals is of insufficient sensitivity and requires an additional ‘master signal’ similar to adaptive immune cells (28). However, whereas we could show that a combined loss of NKG2D, NCR1 and CD16 resulted in a significant reduction in the ability of NK cells to control viral infection and non-hematopoietic tumors, their functionality was not abrogated completely. Instead, loss of these receptors was associated with differential expression and sensitivity to activation by other activating receptors. Exception is CD16-mediated ADCC which cannot be compensated by the expression or sensitivity of other activating receptors (29). Thus, our findings show no evidence that NK cells require a ‘master’ signal for their activation. Instead, non-paired activating NK cell receptors are an integral part of the activation-balance that controls NK cell activity.

A remarkable observation was that TKO NK cells were hyperresponsive to NK1.1 stimulation, which was also observed in CD16-deficent NK cells. CD16 and NK1.1 both use the adaptor molecule FcRγ to mediate intracellular signaling (30, 31). A lack of CD16 may therefore lead to higher availability of FcRγ to NK1.1 and a consequently higher ability to signal downstream. This model of adaptor sequestration was previously observed for NKG2D as animals deficient for this receptor show specific hyperreactivity through NCR1 and CD16 (13). NKG2D sets an activation threshold for NCR1 by increasing CD3ζ protein levels in a mechanism that depends on SLAP-1 during NK cell development (14). In addition to adapter sequestration, non-paired NK cell receptors appear to calibrate signal sensitivity through differential regulation of other surface receptors. Previously, studies have found that deficiency of NKG2D leads to higher expression of DNAM-1 (32). We also find that triple deficiency results in changes in the DNAM-1/TIGIT balance, even though the functional implication of these alterations is still unclear. These findings further elucidate the plasticity of mechanisms regulating NK cell activation, which is regulated both at the receptor level and adaptor molecules mediating their responsiveness. This explains why NK cells appear to have similar activation thresholds, despite having a different receptor repertoire.

Surprisingly, whereas TKO NK cells isolated from B16 melanoma cells had a reduced lytic potential, the same cells showed increased granzyme B accumulation when isolated from MCMV infected tissues. A possible explanation for this discrepancy may be provided by the different inflammatory micro-environment generated under these conditions. Engagement of activating receptors such as NKG2D promotes the lytic capacity of NK cells (33). However, the tumor micro-environment actively suppresses granzyme production by NK cells through induction of inhibitory proteins such as PD-1L (34). This would imply that in TKO mice, both production and release of granzymes are inhibited in TKO mice. Indeed, TKO NK cells show a more exhausted phenotype, which may contribute to their reduced ability to control tumors *in vivo*. In contrast, viral infection is characterized by a strong increase in type I interferons in affected tissues and these cytokines are well known to promote granzyme B production (35). Thus, under these conditions, granzyme B production is promoted, but its release is prevented in TKO mice, leading to its accumulation in the cell.

NK cells pose an attractive mediator of cancer immunotherapy. To prevent recognition of neoantigens by CD8 T cells, tumor cells tend to downregulate self-markers such as HLA-I molecules (36). Moreover, stress caused by the oncogenic process results in their upregulation of ‘induced self’ ligands (37). As a result, cancer cells become susceptible to NK cell mediated control and immunotherapy based on these cells is therefore of immediate interest. Indeed, several clinical trials suggest that NK cells are suited for use in allogeneic therapy without major adverse events. Most NK based immunotherapies are based on the overexpression of chimeric antigen receptors (CARs). However, whereas some successes have been reported, not all patients respond to this type of therapy (38–41). Deeper insight into the biological base of what influences the response of the patient is critical in answering these questions. Our data implies that if one modulates the surface expression of activating receptors, alternations in the expression of other receptors occur in order to preserve the NK cell activation threshold. So, overexpressing a CAR receptor is likely to lower expression of other activating receptors and their adaptors or induce inhibitory receptor expression, which leads to a potential failure of NK cells to respond to tumor targets. Further research into the regulation of NK activity and compensation processes is needed to harvest the full therapeutic potential of NK cells.

Taken together the data presented here show a remarkable compensatory capacity of NK cells to cope with the loss of even three major activating receptors. These findings show that there is still a big gap in our understanding of how NK cells get activated and that a better understanding of NK cell plasticity and mechanisms underlying their activation is crucial for the development of efficient anti-cancer therapies based on these cells.

## 4 Materials and methods

### 4.1 Mice

Mice were strictly age- and sex-matched within experiments and were held in SPF conditions and handled in accordance with institutional, national and/or EU guidelines. Mice used in experiments were between 6 and 12 weeks of age. *Klrk1^−/−^* mice were produced in our laboratory by gene targeting the Klrk11 locus in the C57BL/6 genetic background (13). Wild-type C57BL/6 (strain 000664) and *FcγRIIIa* ^-/-^ (strain 003171) mice were from the Jackson Laboratory. *Ncr1* ^gfp/gfp^ mice were kindly provided by O. Mandelboim (Hebrew University Hadassah Medical School). *FcγRIIIa* ^-/-^ *Klrk1* ^-/-^ *Ncr*1^gfp/gfp^ were generated by interbreeding *Klrk1^-/-^*, *Ncr1^gfp/gfp^* and *FcγRIIIa* ^-/-^ mice. Permission for our experiments was given by Ethical Committee of the Faculty of medicine, University of Rijeka and Croatian Ministry of Agriculture, veterinary and Food Safety Directorate (UP/I -322-01/19-01/64,525-10/0543-19-4).

### 4.2 Construction of chimeras

C57BL/6 Ly^5.1/5.1^ mice were lethally irradiated with 9.5 Gy and 1 d later received 5–10 × 10^6^ bone marrow cells isolated from C57BL/6 Ly^5.1/5.2^ and *FcγRIIIa* ^-/-^ *Klrk1* ^-/-^ *Ncr*1^gfp/gfp^ Ly^5.2/5.2^ mice. Recipients were analyzed 8 wk after transfer. Recipients were given antibiotics (Enroxil 10%; Krka) diluted in a 1:1000 ratio with drinking water for 8–10 d following irradiation.

### 4.3 Cells

B16 cell line was purchased from the American Type and Culture Collection (ATCC). Cells were cultured in complete DMEM, supplemented with 10mM HEPES (pH 7.2), 2mM L-glutamine, 10^5^ U/L Penicillin, 0.1 g/L Streptomycin, and 10% FCS. RMA-S and YAC-1 cells were cultured in complete RPMI 1640, supplemented with 10mM HEPES (pH 7.2), 2mM L-glutamine, 10^5^ U/L Penicillin, 0.1 g/L Streptomycin, and 10% FCS. Cells were maintained in incubator at 37°C, 5% CO_2_.

### 4.4 Tumor models

#### 4.4.1 B16 melanoma

Mice received 10^5^ or 10^6^ B16 cells i.v. or 2 x10^5^ s.c. B16 cells, clone F10 (B16) and survival (reaching of human end points) or tumor growth was followed respectively. Digital caliper was used to measure tumor size. Animal were sacrificed when the tumor reached 1 cm^3^. In these experiments we used 10 mice per group and experiments were repeated at least two times.

#### 4.4.2 RMA-S lymphoma

Mice received 10^5^ RMA-S cells i.v or s.c and survival (reaching of human end points) or tumor growth was followed respectively. Digital caliper was used to measure tumor size. In these experiments we used 10 mice per group and experiments were repeated at least two times.

### 4.5 *In vitro* analysis of NK cells

For NK killer assay B16/YAC-1/RMA-S cells were labeled with proliferation dye eFlour450 (eBioscience) and co-cultured with C57BL/6J or *FcγRIIIa* ^-/-^ *Klrk1* ^-/-^ *Ncr*1^gfp/gfp^ splenocytes, respectively, at an effector to-target ratio adjusted to the number of NK cells. After 4h co-culture in a 5% CO_2_ atmosphere at 37°C, specific lysis was determined in triplicate by flow cytometric analysis as measured by Fixable Viability Dye eFluor780 (eBioscience) incorporation. Spontaneous death of cells was determined in wells containing targets only (42). In co-cultivation assays for cytokine production analysis, splenocytes from WT or *FcγRIIIa* ^-/-^ *Klrk1* ^-/-^ *Ncr*1^gfp/gfp^ mice were co-cultured with B16/YAC-1/RMA-S cells in a 1:1 ratio overnight at 5% CO_2_ at 37°C in addition to IL-2 (100U/mL, R&D Systems), IL-12 (50 pg, R&D Systems) and addition of Brefeldin A (eBioscience) for the last 4h. IFNγ production by NK cells was analyzed by flow cytometry. For *in vitro* NK cell stimulations and analysis of cytokine production 5 x 10^5^ splenocytes were stimulated for 4h at 5% CO_2_ atmosphere and 37°C in addition to IL-2 (100U, Preprotech) and Brefeldin A (eBioscience). For stimulation through specific activating receptor 10 µg/mL of antibody in PBS was pre-coated on an ELISA plate (αLy49H (3D10), αNK1.1 (PK136)). Cytokines were added in suspension: IL12 (10ng/mL, R&D Systems), IL18 (20ng/mL, R&D Systems), IFNβ (20ng/mL, R&D Systems) as well as phorbol-12-myristate-13-acetate (PMA) plus ionomycin. For the proliferation assay, NK cells were enriched from splenocytes using biotinylated DX5 antibodies, streptavidin-coated beads and magnetic cell sorting (Milteny) and then cultured in the presence of IL15 (50ng/mL, R&D Systems) for 4 days. In experiments of *in vitro* NK cell analysis we used minimally 5 mice per group and experiments were repeated two to three times.

### 4.6 Flow cytometry

Cells were pretreated with Fc block (clone 2.4G2, produced in-house). To-pro3 (life Technologies) or Fixable Viability Dye (eBioscience) was used to exclude dead cells. Cells were stained and analyzed in PBS containing 1% BSA and NaN3 with antibodies listed below. For intracellular staining, permeabilization and fixation of cells was done with the Fix/Perm kit (BD Biosciences). Cells were measured on a FACSVerse, FACSAria flow cytometer (BD Biosciences) or MACSQuant (Milteny), and data were analyzed using FlowJo v10 software (Tree Star, Ashland, OR). For flow cytometry, we used monoclonal antibodies to mouse CD3e (145-2C11), CD45.1(A20), CD45.2 (104), NK1.1 (PK136), CD49b (DX5), Ly49H (3D10), Ly49D (4e4), IFNγ (XMG1.2), TNFα (MP6-XT22), NKG2D (CD314) (CX5), CD122 (TM-b1), CD27 (O323), Nkp46 (CD335), DNAM-1 (CD226), TIGIT (MBSA43), Ly49I (YLI-90), Ly49A/D (12A8), CD43 (84-3C1), KLRG1 (2F1), Ly-6A/E (Sca1), Ki67 (SolA15), EOMES (Dan11mag), CD69 (FN50), CD49a (Integrin alpha 1)(TS2/7), CD107a (LAMP-1), CD200R (OX108), Perforin (eBioOMAK-D), Granzyme B (NGZB), (CD11b (M1/70), TIM-3 (CD336)(RMT3-23), LAG-3 (CD223)(3DS223H) and c-kit (CD117)(ACK2) from eBioscience. For hematopoietic stem cell staining, bone marrow cells were stained with Abs against CD4 (GK1.5), CD8 (53-6.7), B220 (RA3-6B2), Gr-1 (RB6-8C5), CD11b (M1/70), TER119 (Ter-119) were used to exclude Lin^+^ cells. Abs were purchased from eBioscience or BD Biosciences.

### 4.7 MCMV infection

The tissue culture grown mCMV C3XR129 was produced in mouse embryonic fibroblasts according to standard protocol. The mice were either treated with αNK1.1 (PK136, 250µg/mouse) or PBS 24h before i.v. injection of 2 x 10^5^ PFU of C3XR129 MCMV. Mice were sacrificed at two time points: one and a half day, and three and a half days. Viral titers were determined in spleens, liver and lungs by standard virus plaque assay. In these experiments 4-6 mice per group were used and experiment was repeated two times.

### 4.8 Quantitation and statistical analysis

To analyze statistical significance, we used Student’s t-test and ANOVA, with Bonferroni’s post-test correction for multiple comparisons. To assess survival rates, the Kaplan–Meier model was used followed by Log-rank (Mantel-Cox) test for pairwise group comparisons. Statistical significance is defined as: * p < 0.05; ** p < 0.01; *** p < 0.001.

## Data availability statement

The original contributions presented in the study are included in the article/**Supplementary Material**. Further inquiries can be directed to the corresponding author.

## Ethics statement

Permission for our experiments was given by Ethical Committee of the Faculty of medicine, University of Rijeka and Croatian Ministry of Agriculture, veterinary and Food Safety Directorate (UP/I -322-01/19-01/64,525-10/0543-19-4).

## Author contributions

VI carried out most of the experiments and analyzed data. VJ, ML performed and analyzed experiments. VJ directed the research. VI, VJ, FMW and BP designed experiments and wrote the paper. All authors contributed to the article and approved the submitted version.

## Supplementary material

Supplementary Figure 1 Shows lymphocytes of TKO NK cells, four stages of maturation of NK cells from *Klrk1^-/-^/FcRγIIIa^-/-^/*Ncr1^gfp/gfp^ mice as well as KLRG1 expression.

Supplementary Figure 2 Shows *in vitro* stimulation with PMA/IONO and NK1.1, of *Klrk1^-/-^/FcRγIIIa^-/-^/*Ncr1^gfp/gfp^ NK cells.

Supplementary Figure 3 Shows survival curve of *Ncr1^gfp/gfp^* and TKO mice after injection of 10^5^ B16 cells i.v.

Supplementary Figure 4 Shows viral titers in the lungs of C57BL/6J/*Klrk1^-/-^/FcRγIIIa^-/-^/*Ncr1^gfp/gfp^ mice, viral titer in the spleen and liver of TKO and WT mice after NK1.1 depletion and the expression of CD69, KLRG1, Granzyme B, TIM-3, TIGIT and LAG-3.
