# Supplementary material for: Largely preserved functionality after the combined loss of NKG2D, NCR1 and CD16 demonstrates the remarkable plasticity of NK cell responsiveness
Source: Front Immunol. 2023 Jul 13;14:1191884. doi: 10.3389/fimmu.2023.1191884 (PMC10374020; doi:10.3389/fimmu.2023.1191884)
Supplement: Supplementary Figure 1 — Shows lymphocytes of TKO NK cells, four stages of maturation of NK cells from Klrk1-/-/FcRγIIIa-/-/Ncr1gfp/gfp mice as well as KLRG1 expression. [file DataSheet_1.pdf]

Supplementary Material

Largely preserved functionality after the combined loss of NKG2D, NCR1 and CD16 demonstrates the remarkable plasticity of NK cell responsiveness.

Vanna Imširović[1], Maja Lenartić[1], Felix M. Wensveen[1], Bojan Polić[1] and Vedrana Jelenčić[1]

[1] Department of Histology and Embryology, Faculty of Medicine, University of Rijeka, Rijeka, Croatia

*** Correspondence:**
Vedrana Jelenčić
vedrana.jelencic@uniri.hr

1 Supplementary Figures and Tables

1.1 Supplementary Figures

Supplementary Figure 1.

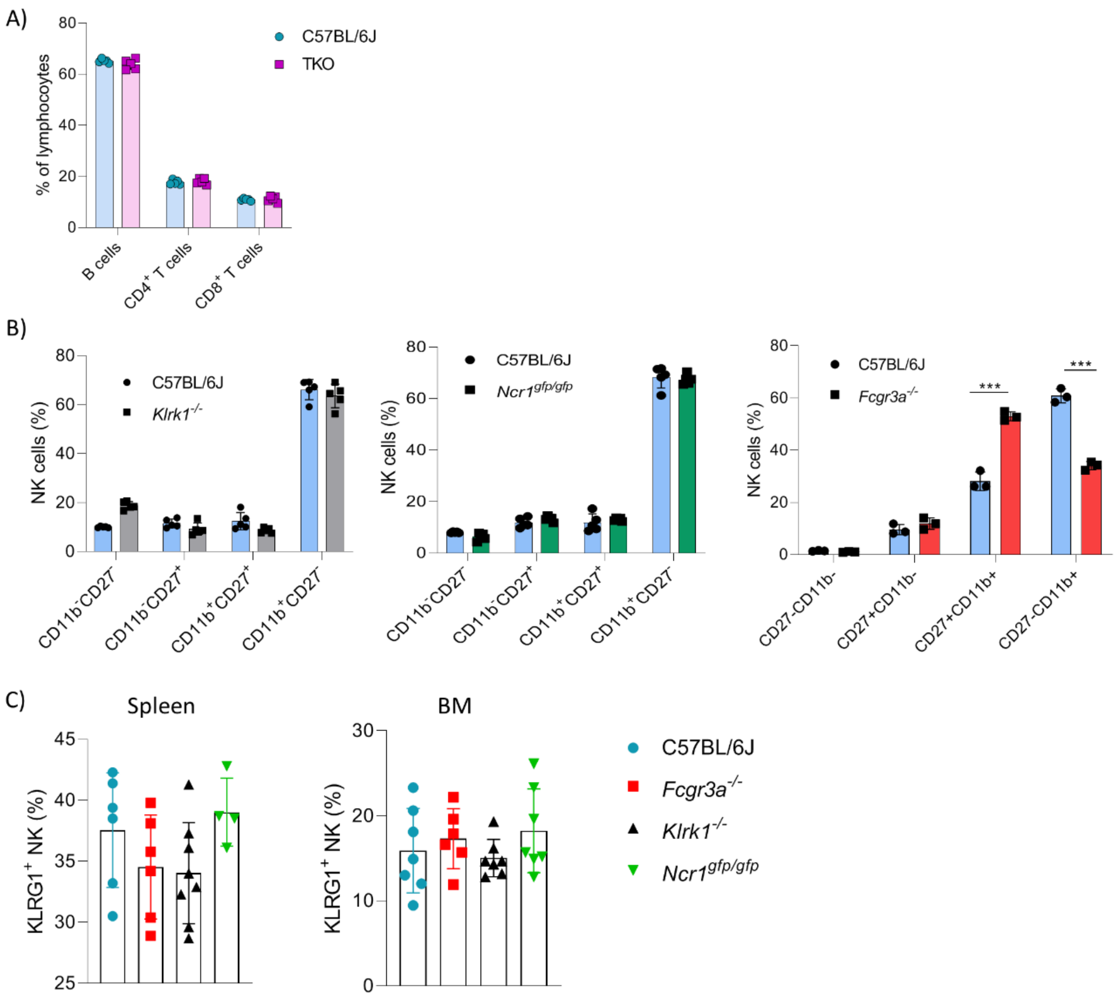

Supplementary Figure 1

(**A**) Frequency of B cells, $CD4^{+}$ and $CD8^{+}$ T cells in the spleen of WT (C57BL/6J) and $Fc\gamma r3a^{-/-}$ $Klrk1^{-/-}Ncr1^{gfp/gfp}$ (TKO) mice (n = 5 mice per group). (**B**) Percentages of CD11b, CD27 expression on NK cells in the spleen of WT, $Klrk1^{-/-}$, $Ncr1^{gfp/gfp}$ and $Fc\gamma r3a^{-/-}$ mice (n = 3-5 mice per group). (**C**) Percentages of $KLRG1^{+}$ NK cells in the spleen and BM (n = 4-7 mice per group). Each symbol represents an individual mouse. Data shown represent two independent experiments. Mean ± SD is shown. ***$p < 0.001$. Unpaired Student's t-tests (two-tailed) (A, D) or Two-way ANOVA (B) were used to calculate these values.

Supplementary Figure 2.

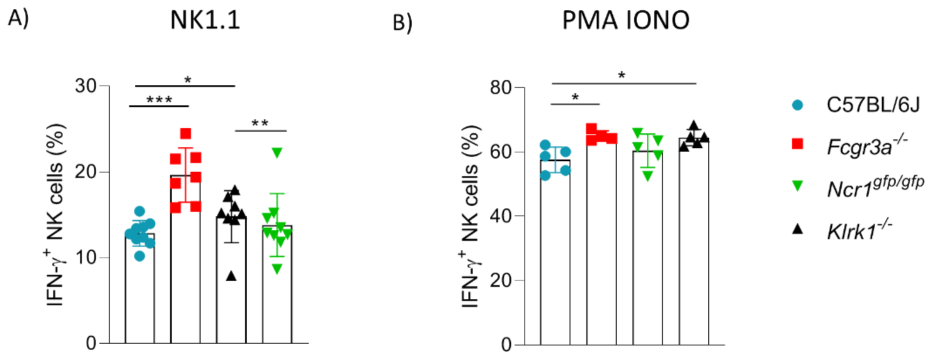

Supplementary Figure 2

(**A-B**) NK cells from WT, *Fcγr3a*$^{-/-}$, *Klrk1*$^{-/-}$ and *Ncr1*$^{gfp/gfp}$ mice (n = 5-9 per mice group) were stimulated for 4 h through the NK1.1 receptor by mAb or with PMA/IONO and IFN-γ production was analyzed after 4 h. Data shown represent two independent experiments. Mean ± SD is shown. *p < 0.05, **p < 0.01 and ***p < 0.001. One-way ANOVA was used to calculate these values.

Supplementary Figure 3.

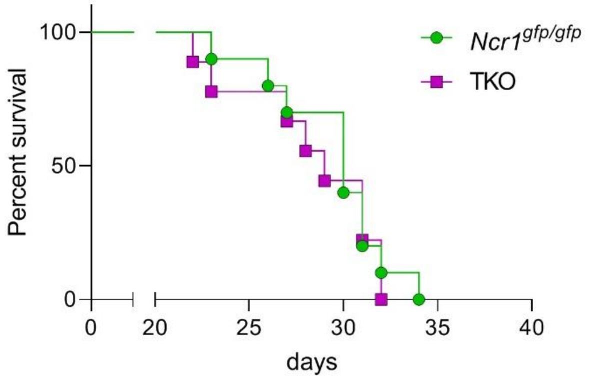

Supplementary Figure 3

Graph shows survival curve of Data shown represents one independent experiment. Survival curve was analyzed by the Kaplan-Meier model followed by log-rank (Mantel-Cox) test (two-tailed).

Supplementary Figure 4.

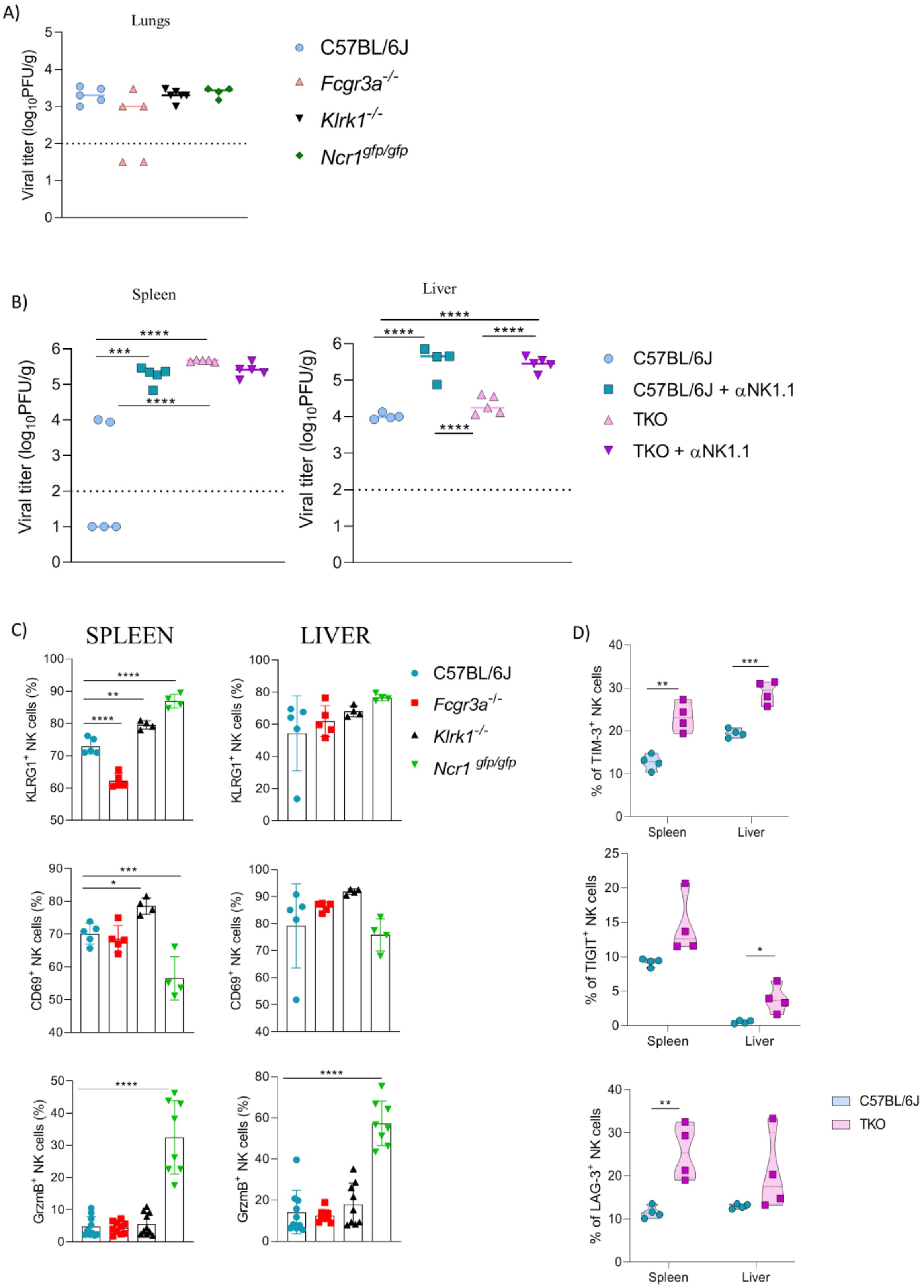

Supplementary Figure 4

(**A**) Virus titers in the lungs of WT, *Fcγr3a*$^{-/-}$, *Klrk1*$^{-/-}$ and *Ncr1*$^{gfp/gfp}$ mice (n = 4-5 mice per group). (**B**) TKO and WT mice (n = 5 mice per group) were treated with anti-NK1.1 one day before the infection with WT MCMV (2 x 10^5 PFU intravenously); viral titers were assessed in the spleen and liver. Percentages of NK cells expressing KLRG1, CD69 and Granzyme B in the spleen (**C**) and liver of WT, *Fcγr3a*$^{-/-}$, *Klrk1*$^{-/-}$ and *Ncr1*$^{gfp/gfp}$ mice (n = 4-5 mice per group). (**D**) Percentages of NK cells expressing TIM-3, TIGIT and LAG-3, isolated from the spleen and liver of WT and TKO mice (n = 4 mice per group). Data shown represent one out of two independent experiments. Mean ± SD is shown for the presented data. *$p < 0.05$, **$p < 0.01$, ***$p < 0.001$ and ****$p < 0.0001$. Viral titers were analyzed using Kruskal–Wallis test. One-way ANOVA (C-D) and Unpaired Student’s t-test (two-tailed) (E-F) were used to calculate these values.
